# Ichthyosis vulgaris: An updated review

**DOI:** 10.1002/ski2.187

**Published:** 2022-11-25

**Authors:** Huda Jaffar, Zobia Shakir, Gaurav Kumar, Iman Fatima Ali

**Affiliations:** ^1^ Dow University of Health Sciences Karachi Pakistan

## Abstract

Ichthyosis vulgaris is an inherited, non‐syndromic form of ichthyosis that presents with skin problems. Making up more than 95% cases of ichthyosis, ichthyosis vulgaris is caused by heterozygous loss‐of‐function mutation of the filaggrin gene, raising the fragility and permeability of the stratum corneum. It typically presents in infancy as xerosis, skin lesions, keratosis pilaris, palmoplantar hyper linearity, scaly dermatosis, and erythroderma, clearly identifiable by age 5. Although majority of patients have a normal lifespan, possible complications include a vitamin D deficiency and auditory problems due to scaling in the ears, besides a drop in quality of life due to dermatological changes. Urea‐based creams with 10% urea, ceramides, and other ceramides are often the first line therapy in ichthyosis vulgaris. There is no known curative treatment for ichthyosis vulgaris, but lifelong treatment can alleviate the symptoms. Urea‐based creams are highly therapeutic, whereas ammonium lactate 12% lotion with a physiological lipid‐based repair cream can help with scaling and dryness. There is also evidence in favour of propylene glycol solutions. Risankizumab, an anti‐interleukin‐23 drug, and enhancement of natural moisturizing factors are also two highly promising solutions that require additional research. This review aims to provide updates on the manifestation, evaluation, and treatment of ichthyosis vulgaris.

## Graphical abstract

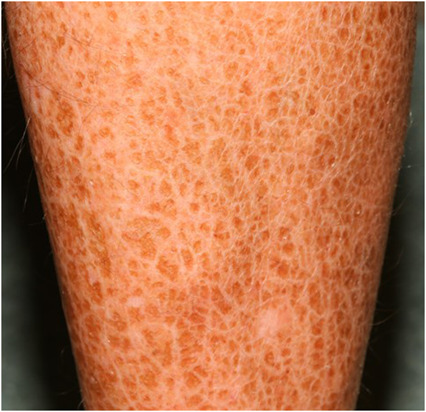

Ichthyosis Vulgaris, what is known up till now, and what is the future looking like?

1

**What's already known about this topic?**

- Ichthyosis vulgaris is an inherited skin disease due to loss‐of‐function mutations of the filaggrin gene.
- Several publications have discussed the disease's clinical manifestations and treatment strategies to improve prognosis over the years.

**What does this study add?**

- We provide a concise overview of the clinical presentation of the disease along with its treatment options.
- Promising treatment options that need further exploration include urea‐based creams, propylene glycol, and Risankizumab, an anti‐interleukin‐23 drug.

## 2 INTRODUCTION

Ichthyoses are a group of cutaneous disorders of keratinization characterized by dry, rough skin with prominent scaling.^1^ Ichthyosis vulgaris is an inherited form of ichthyosis caused by loss‐of‐function mutations in the filaggrin gene (FLG).^1^, ^2^ The clinical presentation of ichthyosis vulgaris includes xerosis, keratosis pilaris, palmar hyperlinearity and a predisposition to atopic disorders.^2^, ^3^ Ichthyosis vulgaris exhibits an autosomal semi‐dominant pattern of inheritance with a milder phenotype in heterozygotes^4^, ^5^ and an estimated prevalence of around 1:100 to 1:250^1^ making it the most frequently occurring form of ichthyosis.^6^, ^7^ The age of onset of ichthyosis vulgaris is during infancy and most patients exhibit clear clinical manifestations by the age of 5.^4^

## 3 EPIDEMIOLOGY

With a prevalence of around 1:100 to 1:250, ichthyosis vulgaris is the most common type of ichthyosis.^1^ Accounting for more than 95% cases of ichthyoses, this disease manifests in infancy during the first year of life or early childhood, often at birth.^7^ There are no notable significances in sex or ethnicities and the disease is observed to improve with age.^4^

The frequency of FLG mutation is 8%–10% among the general population.^8^ The spectrums of filaggrin mutations vary according to ancestral populations with R501X and 2282del4 being the most common ones among Europeans.^9^, ^10^, ^11^ These are less widespread in Asian populations where 3321delA is most common (absent in Europe), whereas E2422X is observed in both European and Asian populations.^12^, ^13^, ^14^, ^15^ Mutations R224x and S3247X are also present in Europe, albeit at a lower frequency,^16^ while 3331delA is also widespread in Japan.^17^

Since loss‐of‐function mutations in FLG are a major genetic predisposing factor for atopic dermatitis (AD),^2^, ^5^, ^10^ 2.5%–37% patients with AD possess clinical evidence of ichthyosis vulgaris.^18^ A statistically significant (*p* < 0.001) association is also seen between longer eyelashes and hyperlinearity (*p* = 0.019), a major symptom of ichthyosis vulgaris.^18^

## 4 AETIOLOGY AND PATHOGENESIS

Ichthyosis vulgaris is caused by heterozygous loss‐of‐function mutations in the FLG^5^ which result in absolute or relative deficiency of filaggrin, disrupting the epidermal barrier. The FLG is found in the epidermal differentiation complex on chromosome 1q21.^6^ FLG encodes profilaggrin, a complex, highly phosphorylated polypeptide rich in histidine. Profilaggrin is an important component of the F‐type keratohyaline granules found in the stratum granulosum^4^, ^8^ and serves as the precursor molecule for the synthesis of filaggrin.^5^, ^19^ During epidermal terminal differentiation, profilaggrin is dephosphorylated and cleaved by serine proteases into 10–12 monomeric filaggrin protein molecules in the stratum corneum.^5^, ^19^ Filaggrin is a 37 kDa multi‐functional, histidine‐rich, and insoluble structural protein^8^ that is an essential component of the cornified cell envelope^20^, ^21^, ^22^, ^23^ Filaggrin flattens^24^, ^25^ by aggregating, aligning, and cross‐linking the bundles of keratin intermediate filaments during differentiation,^26^ enhancing the mechanical resilience and integrity of the stratum corneum's cytoskeleton. Since the stratum corneum prevents water loss through the epidermis and entry of allergens, toxic chemicals, and pathogens,^24^, ^27^ filaggrin plays a key role in the mechanical and protective function of the skin barrier. In the stratum corneum, FLG is broken down into substances like trans‐urocanic acid (UCA) and pyrrolidone carboxylic acid (PCA)^6^ which are natural moisturizing factors (NMF) that hydrate the skin,^28^ maintain acidic pH,^28^ photosensitivity,^20^ skin elasticity,^20^ and limit overgrowth of microbes.^28^

FLG deficiency raises the fragility and permeability of the stratum corneum.^29^ The disruption of the mechanical barrier increases trans epidermal water loss and worsens the immunological barrier against allergens and microorganisms. Water loss exacerbates skin elasticity, while the change in pH affects the keratinocyte differentiation process.^1^ The patient is more prone to allergens, thereby increasing risk of AD, hand eczema, allergic contact dermatitis, and irritant contact dermatitis.^30^, ^31^, ^32^, ^33^ AD patients with FLG mutations experience a more severe form of the disease, coupled with a higher incidence of *Staphylococcus aureus* and herpes virus infections, asthma, and allergic sensitization than AD patients without FLG mutations.^19^

Ichthyosis vulgaris is an autosomal semidominant condition with incomplete penetrance, about 83%–96%,^17^ and variable expressivity, such that disease severity can vary even within affected families.^20^ FLG mutations cause ichthyosis vulgaris in both Caucasian and Asian populations, but they tend to be population specific with different and sometimes mutually exclusive mutations between these groups. The prevalence of ichthyosis vulgaris in darkly pigmented populations appears to be low, but more studies are required to confirm these observations.^17^ The FLG gene consists of three coding exons [Presland]. Exon 1 (15bp) contains 5^0^UTR sequences and is non‐coding. Transcription and translation start at Exon 2, and the highly repetitive Exon 3 (>12 kb) codes for the majority of the profilaggrin polypeptides. Overlapping long‐range PCR method to sequence the entire code of FLG gene in Iranian patients showed no alternation in exon 1 and 2 sequences during mutation.^19^ In this study, 43 previously reported single nucleotide polymorphisms (SNPs) and two novel variants (p.S417S and p.D1291N) were observed but all of them were benign. The most common FLG mutations in the European population are the null mutations: R501X and 2282del4.^19^ Additional 60 loss‐of‐function mutations have also been identified,^34^ such as 3321delA among the Asians, E2422X among both Europeans and Asians,^12^, ^13^, ^14^, ^15^ and low‐frequency mutations like R244x and S3247X.^16^ The frequency and type of these mutations vary among populations, and they all lead to loss of filaggrin expression. Another mutation, p. Arg2037Ter in the heterozygous condition was previously identified as pathogenic in databases^35^ with a penetrance of 90%. This nonsense mutation introduces a premature stop codon in the transcript of the mutant at position 2037, resulting in a truncated protein. Twenty‐five variants in FLG were also identified in a Chinese study,^3^ of which 9 (36%) were not previously reported. Targeted next‐generation sequencing has identified nonsense and frame‐shift variants in FLG mutations. This included 11 frame‐shift variants and 14 nonsense variants, including the most common 3321delA.^3^

## 5 HISTOPATHOLOGY

Histopathological features of ichthyosis vulgaris include a mild orthokeratotic hyperkeratosis (with or without hypogranulosis or agranulosis) and considerably reduced or absent keratohyalin granules in the epidermis.^4^, ^36^, ^37^ The granules are also smaller in size,^36^ and the stratum granulosum is either reduced or completely absent.^1^ Inflammatory infiltrate is also absent.^4^ Additionally, three dimensional organotypic cultures reveal a lower (not total disappearance) corneodesmosome density, defective loading, and premature secretion of lamellar bodies' contents into the extracellular spaces of stratum granulosum on an ultrastructural level.^25^, ^37^ This incomplete processing of lamellar material results in an abnormal lamellar bilayer structure which is another hallmark feature of ichthyosis vulgaris.^25^

Infrared spectroscopy further reports reduced stratum corneum lipid organization,^38^, ^39^ whereas a hyperplastic epidermis^6^ due to increased proliferation of keratinocytes, and weak tight junctions is also observed in the stratum corneum.^25^ This hyperplasia is what results in continuous, widespread scales on the body.^40^ The morphology of the suprabasal epidermal layers, on the other hand, is minimally affected, if at all.^41^

Although filaggrin monomers damage the keratin filament network, studies on ichthyosis vulgaris report either normal filaments^36^, ^42^ or their perinuclear retraction.^25^ In fact, the amounts of KRT 5, 6, and 1 are unaffected between ichthyosis vulgaris keratinocytes and healthy cells.^42^

## 6 CLINICAL MANIFESTATIONS

**FIGURE 1:**
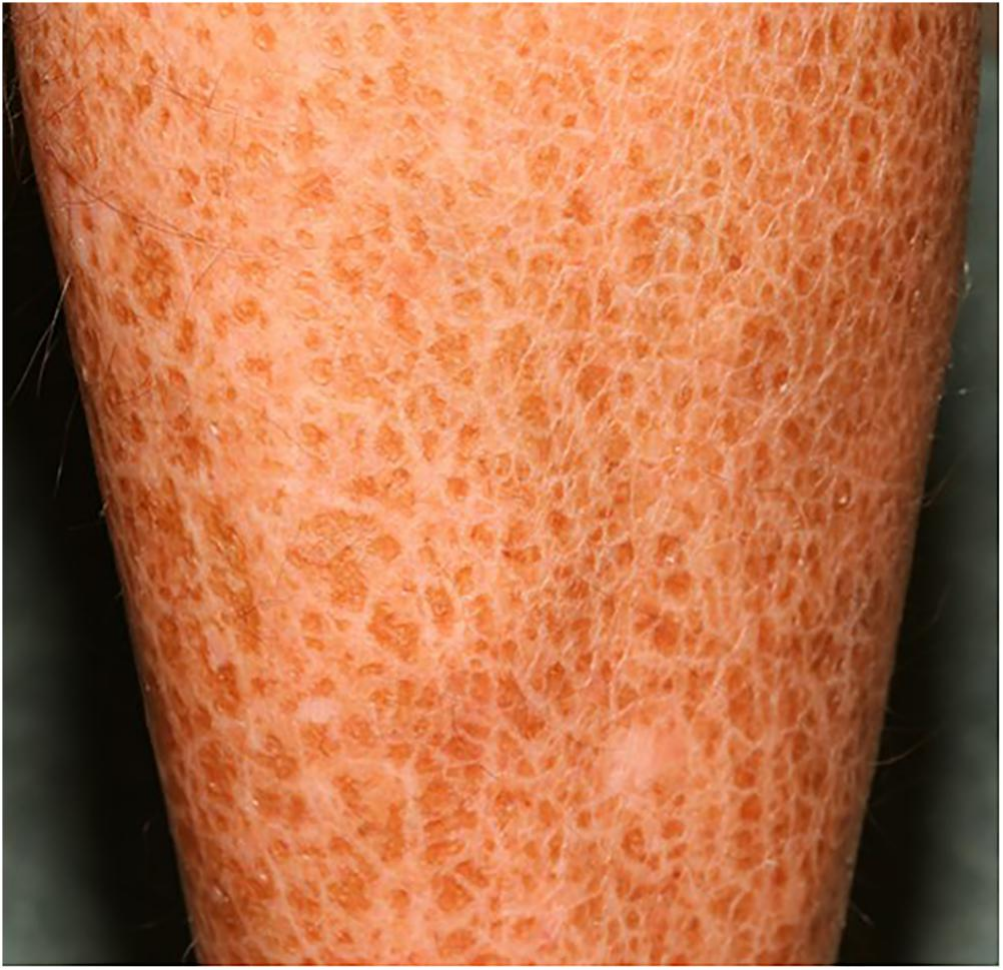
Dry, scaly skin of an ichthyosis vulgaris patient

**FIGURE 2:**
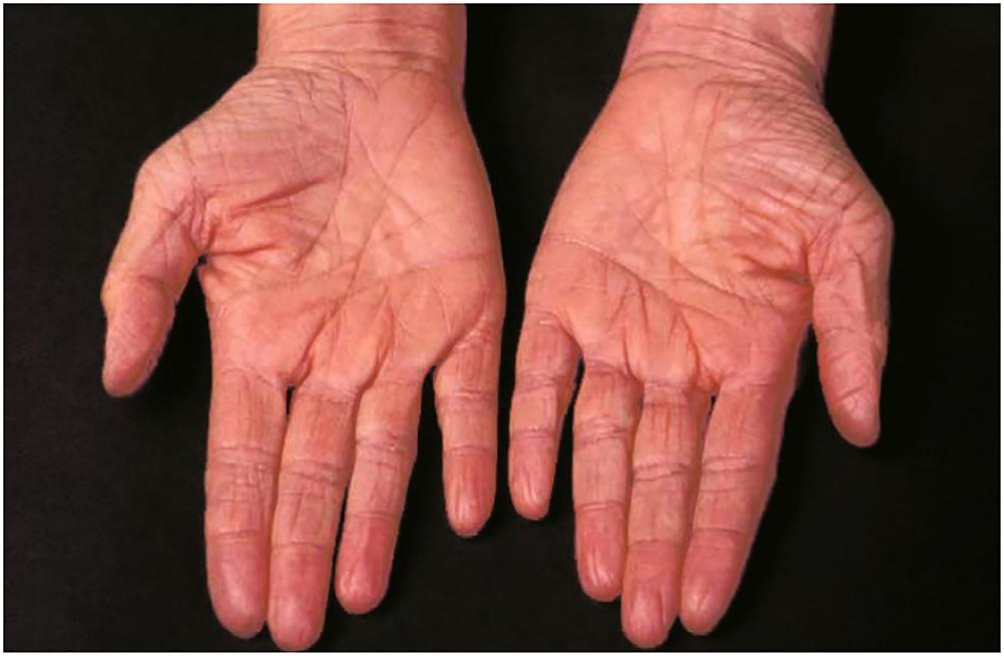
Palmar hyperlinearity in a 40‐year‐old ichthyosis vulgaris patient

The term ‘ichthyosis’ is derived from the Greek ‘ichthys’ meaning fish, because this condition is characterized by fish‐like dry, scaly, and thickened skin (hyperkeratosis) (Figure 1).^43^, ^44^ Most common manifestations of ichthyosis vulgaris include xerosis (dry skin), partial or total skin lesions, keratosis pilaris and palmoplantar hyperlinearity (Figure 2)^17^, ^45^ along with generalized scaly dermatosis without the component of erythema^4^ and a varying degree of erythroderma.^1^ Despite the dry skin, however, ichthyosis vulgaris patients do not exhibit signs of overt inflammation.^37^

These clinical symptoms are exhibited at infancy, with the majority of sufferers having fully displayed manifestations by age 5. However, the severity of clinical features varies from mild xerosis in some individuals to large adherent scales (lizard skin) in others.^4^ In some individuals, symptoms are mild enough to escape a diagnosis,^1^ whereas hyperkeratotic plaques with fissures on palmoplantar skin is a severe manifestation that threatens the neurovascular integrity of digits and/or limbs if no surgical intervention is sought.^9^, ^17^, ^46^ Homozygous manifestations are severe, with large, centrally adherent scales on the lower limb and trunk. Additionally, the scaling exacerbates during the winter before improving again in the summer and humid climates.^4^ Homozygous FLG mutation carriers have a complete absence of filaggrin expression which presents as a stable skin phenotype with chronic presence of ichthyosis vulgaris features. In a study assessing a family of 15, heterozygotes displayed a mild phenotype with incomplete penetrate whereas the homozygotes possessed widespread and obvious scaling and marked hyperlinearity.^5^ Both the trans epidermal water loss (TEWL) and the number of differentially expressed genes in the epidermis are higher in patients with homozygous mutations.^31^

Dry skin, whitish to light brown skin flakes due to peeling^43^ and fine grey or whitish scales^1^ have a primarily extensor distribution.^4^ The extensor surfaces of the upper and lower extremities are the initial target^43^ but the lower abdomen, feet, and soles may also be affected.^9^, ^17^, ^46^ The flexural creases, however, are spared.^4^ There is also no hair loss, changes in the nails, or onychauxis. White seborrhoeic plagues are other common sights on the extremities.^43^

Süßmuth et al stated that since ichthyosis vulgaris is a non‐syndromic form of ichthyosis, there is no extracutaneous involvement of the eyes, ears, olfactory, skeletal, and the nervous systems.^1^, ^43^ However, another study mentioned keratoconus and ectropion as ocular manifestations in a male patient.^47^

Ichthyosis vulgaris has a strong association with atopic disorders such as atopic dermatitis, allergic rhinitis (hay fever), and asthma.^17^, ^48^ Atopic disease is reported in 35%–70% of patients with ichthyosis vulgaris,^49^ with more than 50% of them having atopic dermatitis.^1^ Pruritus, allergic rhinoconjunctivitis, hypohidrosis, heat intolerance, and recurrent infections due to a disturbed skin barrier are other common occurrences.^43^

Ichthyosis vulgaris has also been presented with comorbidities such as x‐linked ichthyosis,^35^ disseminated tinea,^50^ palmoplantar pustulosis (PPP),^51^ and acute intermittent porphyria.^52^ However, the association between ichthyosis vulgaris and any of these diseases has not been proved and could be a coincidence.

## 7 DIFFERENTIAL DIAGNOSES

The differential diagnoses of ichthyosis vulgaris include atopic dermatitis, allergic and irritant contact dermatitis, asteatotic ezema (eczema craqeulé) and even xerosis, which is itself a predisposing factor for the latter.^53^ Other differential diagnoses are lamellar ichthyosis, x‐linked ichthyosis, Harlequin ichthyosis, drug eruptions and Sjogren Larsson syndrome.^4^, ^54^

Acquired ichthyosis is also a differential diagnosis. This is associated with systemic diseases such as Hodgkin lymphoma, sarcoidosis, thyroid disorders, and human immunodeficiency virus infection, and presents in adulthood.^54^

## 8 COMPLICATIONS

The quality of life (QoL) of a person with ichthyosis vulgaris is greatly affected. The noticeable scales in addition with associated erythema, pruritis leading to lichenification and disfiguration,^4^ anhidrosis, and associated secondary infections, dehydration, mineral, and protein loss from the disrupted skin barrier impose hindrance in daily activities and in the social life of the patient.^55^ According to the study, the severity of affected QoL is in the following order: LI > IV > XRI. Adults are affected more than children.^55^

Due to loss of normal skin function, ichthyosis vulgaris can lead to vitamin D deficiency associated with rickets and growth failure in children. Lucia et al. mentioned a study conducted between 10 American children. The study concluded that the children with ichthyosis have nutritional deficiency despite normal intake of calories. This is because loss of nutrients from the disrupted skin increases the calorie requirement than normal.^55^

Furthermore, ocular manifestations are an important common association with ichthyosis vulgaris and other types of ichthyosis. Mohammad A et al. conducted a study in Saudi Arabia which included 11 patients with different types of ichthyosis. Ectropion was stated to be the most common manifestation of both upper and lower eyelids, and this complication was commonly involved with lamellar ichthyosis. Ectropion leads to exposure keratopathy, keratitis, and even corneal perforation which effects visual acuity and is harder to treat. Ectropion itself can be treated via skin graft and has a good prognosis.^47^ Other ocular complications include retinitis pigmentosa in patients with Refsum disease which includes ichthyosis as one of its components. Cataract is also a complication of many types of ichthyosis.^55^

The intense scaling present in ichthyosis vulgaris can collect in the external auditory canal and block it, affecting hearing of the patient.^55^ Huang et al carried out a survey evaluating the types and frequency of ear symptoms and its demographics. She mentioned that patients experienced immense ear pain, itching, and decreased hearing. Hearing loss was reported in 74% adult patients that were older than 18 years and in 55% of patients aged 18 and younger. Almost half of the patients that had abnormal hearing test started using a hearing aid.^56^

A few cases of congenital ichthyoses have been associated with skin malignancies, most commonly squamous cell carcinoma. Association of ichthyosis with melanoma was reported in 3 cases only but in all of these cases patients had non bullous congenital ichthyoses erythroderma. It is suggested that chronic inflammation of the defective skin may lead to carcinogenesis mainly in congenital form. No reports of melanoma with ichthyosis have been published but there is a possibility of this association.^57^

Moreover, a study reported a few cases of disseminated tinea infection in a patient with eczema and ichthyosis vulgaris. The causative agents were found to be Trichophyton rubrum and Trichophyton mentagrophytes. The tinea lesions were widespread and symmetrical, similar in appearance in these cases. There is increasing risk of recurrent tinea in ichthyosis vulgaris patients especially in autosomal recessive congenital ichthyoses because of impaired skin barrier, chronic use of topical corticosteroids in the treatment of ichthyosis vulgaris, and when these patients travel to dermatophytosis endemic countries.^50^

## 9 TREATMENT

Ichthyosis vulgaris has no known curative treatment and the management aims to suppress its symptoms and improve the well‐being of patients.^58^ Treatment mainly includes moisturizing agents containing lactic acid, glycerol, or urea.^45^

Urea, with its moisturizing and keratolytic properties can restore stratum corneum function, hydration and prevent excess keratin accumulation due to which it can be used as a first line therapy in ichthyosis vulgaris. Celleno L et al mention that urea also enhances skin penetration of other drugs, for example, corticosteroids, therefore it can be effectively used in combination therapies.^59^

Clara et al conducted a study where ichthyosis vulgaris patients were given urea‐based emulsions containing 10% urea, ceramides and NMFs to be applied twice daily. After monitoring patients for 30 days, it was concluded that this emulsion not only improved significant amount of dryness, but reduced pruritis and was cosmetically acceptable. Moreover, videodermatoscopy and reflectance confocal microscopy also showed disappearance of scales.^60^ Therefore, urea‐based solutions are very popular in treating xerosis and scales. However, according to Pope et al, it is expensive and hence not reliable because ichthyosis vulgaris requires lifelong continuous treatment.^61^

Drugs are used to reduce keratinization, restore lipid‐based skin barrier, reduce pruritis and prevent superimposed infections. Different studies provided evidence in favour of a number of moisturizing lotions to help restore the lipid‐based skin barrier in ichthyosis vulgaris patients. A study conducted by Bellew et al. proved the effectiveness of combination therapy including ammonium lactate (AL) 12% lotion and physiological lipid‐based barrier repair cream (Ec).^62^ AL is a lactic acid which is part of Natural Moisturizing Factors (NMF) of the skin. Ec includes ceramides, cholesterol, and free fatty acids which are the components of the normal intercellular lipid layer. Both these creams were applied twice daily on a 10‐year‐old boy with a chronic history of ichthyosis vulgaris. Scaling and dryness were completely eradicated after 1 month and there was no return of symptoms with continued use of the formulations.^62^

In another study, Goldsmith et al observed the effectiveness of propylene glycol with occlusion for the treatment of different types of ichthyosis. 40% and 60% aqueous solutions of propylene glycol were given to patients for daily application and were asked to cover the affected areas overnight. It was concluded that almost all the scales could easily be removed during bath after 2 to 3 applications in both x‐linked ichthyosis and ichthyosis vulgaris. 60% dilution was more effective than 40%. After removing the scales, the solution was reapplied. The treatment however seemed unresponsive if application was not followed by occlusion. Intermittent application is necessary to keep skin clear of scales. Propylene glycol solution has no systemic toxicity, it is cheap and easy to use rendering it a reliable option for ichthyosis vulgaris.^63^

Another treatment option is retinoids, specifically Trifarotene which was granted Orphan Drug Designation by FDA for the treatment of congenital ichthyoses.^64^

In another case report, Risankizumab, an interleukin‐23 drug significantly improved both PPP and ichthyosis vulgaris in a 60‐year‐old female patient, rendering it as a possible treatment for ichthyosis vulgaris. She was given Guselkumab first, which is also an interleukin‐23 drug, however that was only effective against PPP.^51^

## 10 PROGNOSIS

In cases of acquired ichthyosis, once the disease that triggers it is treated, there is a possibility of ichthyosis being treated too. Similar to this, if a drug is the cause, reducing the dose of that specific also shows a good prognosis.

The prognosis for inherited cases has proven to be good among majority of patients, leading to a normal lifespan and an improvement in the disease with age. However, treatment is usually continued throughout life.^58^

## 11 CONCLUSION

Ichthyosis vulgaris is an inherited skin disease due to loss of function mutations in FLG gene. There is a lot of data regarding its treatment options which improve quality of life, however, a definitive multidisciplinary treatment approach targeting the skin, along with the patient's mental health has yet to be explored.

## AUTHOR CONTRIBUTIONS

**Huda Jaffar**: Conceptualization (Lead); Data curation (Lead); Methodology (Lead); Supervision (Lead); Writing – original draft (Equal); Writing – review & editing (Lead); **Zobia Shakir**: Conceptualization (Equal); Data curation (Equal); Methodology (Supporting); Writing – original draft (Equal); Writing – review & editing (Equal); **Gaurav Kumar**: Conceptualization (Equal); Data curation (Equal); Methodology (Equal); Writing – original draft (Equal); Writing – review & editing (Equal); **Iman Fatima Ali**: Conceptualization (Equal); Data curation (Equal); Methodology (Equal); Writing – original draft (Equal); Writing – review & editing (Equal).

## CONFLICTS OF INTEREST

The authors declare that they have no conflicts of interest.

## ETHICS STATEMENT

Not applicable.

## Data Availability

The data that support the findings of this study are available from the corresponding author upon request.
